# Bayesian transcriptome assembly

**DOI:** 10.1186/s13059-014-0501-4

**Published:** 2014-10-31

**Authors:** Lasse Maretty, Jonas Andreas Sibbesen, Anders Krogh

**Affiliations:** The Bioinformatics Centre, Department of Biology and Biotech Research and Innovation Centre (BRIC), University of Copenhagen, Ole Maaløes Vej 5, Copenhagen, 2200 Denmark

## Abstract

RNA sequencing allows for simultaneous transcript discovery and quantification, but reconstructing complete transcripts from such data remains difficult. Here, we introduce *Bayesembler*, a novel probabilistic method for transcriptome assembly built on a Bayesian model of the RNA sequencing process. Under this model, samples from the posterior distribution over transcripts and their abundance values are obtained using Gibbs sampling. By using the frequency at which transcripts are observed during sampling to select the final assembly, we demonstrate marked improvements in sensitivity and precision over state-of-the-art assemblers on both simulated and real data. *Bayesembler* is available at https://github.com/bioinformatics-centre/bayesembler.

**Electronic supplementary material:**

The online version of this article (doi:10.1186/s13059-014-0501-4) contains supplementary material, which is available to authorized users.

## Background

The massive throughput of second-generation sequencing technologies is rapidly changing our ability to explore complex transcriptomic landscapes as it can reveal both sample-specific transcript variants and their abundances (i.e. expression levels). However, due to the combination of alternative splicing and the short sequencing fragments characteristic of these methods, it is often not possible to determine directly which exons are linked in splice variants over longer sequence distances. Instead, due to variation in abundance between alternative splice variants, read coverage along exons and splice junctions can be used to infer the most likely exon combinations.

We define transcriptome assembly as the problem of determining the set of expressed transcripts and their abundance levels in a sample from a set of RNA sequencing (RNA-seq) reads. Current assembly algorithms generally proceed by first extracting exon boundary and splice junction information from the RNA-seq reads, which is then used to build a set of splice graphs representing all possible splice variants [1]. This problem can generally be solved efficiently using either a reference-based strategy, where a reference genome is used as a scaffold in splice graph assembly, or by *de novo* construction. Given a graph, the challenge is then to determine which combination of transcripts – represented by paths in the graph – and associated abundances best explains the data. However, as shorter sequencing fragments give rise to larger numbers of putative transcripts, we know *a priori* that we should generally search for solutions that are sparse relative to the total number of paths in the graph. The inference objective is thus to find solutions sufficiently rich to explain both the graph and its read coverage without overfitting by predicting too many transcripts.

The widely used *Cufflinks* [2] method estimates splice variants and their abundances sequentially and solves the former problem by searching for the smallest set of transcripts that can explain the graph guided by splice junction coverage information. However, the use of only local coverage information makes it susceptible to noise and the search for the minimal set of transcripts lacks a biological foundation as more complex solutions may better explain the full graph coverage. More recent assemblers like *IsoLasso* [3], *SLIDE* [4], *CEM* [5] and *iReckon* [6] co-estimate splice variants and abundances using regularisation-based methods. But these approaches achieve sparsity by effectively thresholding transcript abundances and thus implicitly penalise lowly abundant transcripts. The *Mitie* [7] assembler avoids the thresholding approach to regularisation by using a greedy variant of mixed-integer programming, which, however, comes at the risk of only finding suboptimal solutions. Similarly, the *Traph* [8] assembler pursues the simplest possible transcript solution also using a greedy graph-optimisation algorithm. More generally, most assemblers rely on a number of hard-to-tune hyperparameters and heuristic thresholds, which suggests that the methods may not generalise well across, for example, species and RNA-seq protocols. Finally, even under sparse estimation conditions, model identifiability issues and noise may still give rise to uncertainty about the correct combination of expressed transcripts, thus motivating a fully probabilistic approach to the assembly problem.

Here, we present a novel probabilistic approach to transcriptome assembly based on an efficient Gibbs sampling method for inference in a Bayesian model of the RNA sequencing process. By modelling a subset of paths in the graph – or transcript candidates – as having point-zero abundance, the Bayesian formulation allows us to model the prior expectation of the number of expressed transcripts (sparsity) without penalising lowly expressed ones. The frequency at which transcript candidates are observed to have positive abundance in a set of Gibbs samples then serves as a confidence metric for each transcript. These confidence estimates are in turn used to determine the final assembly and further provide a means for prioritising assembled transcripts for downstream validation. Our method is implemented in C++ as a complete transcriptome assembly package under the name *Bayesembler*.

## Results

We first present an informal overview of our model and inference method; a formal description is provided under Materials and methods. We then compare the performance of our assembler with a panel of state-of-the-art transcriptome assembly methods on a number of different datasets.

### The Bayesembler

**Figure 1:**
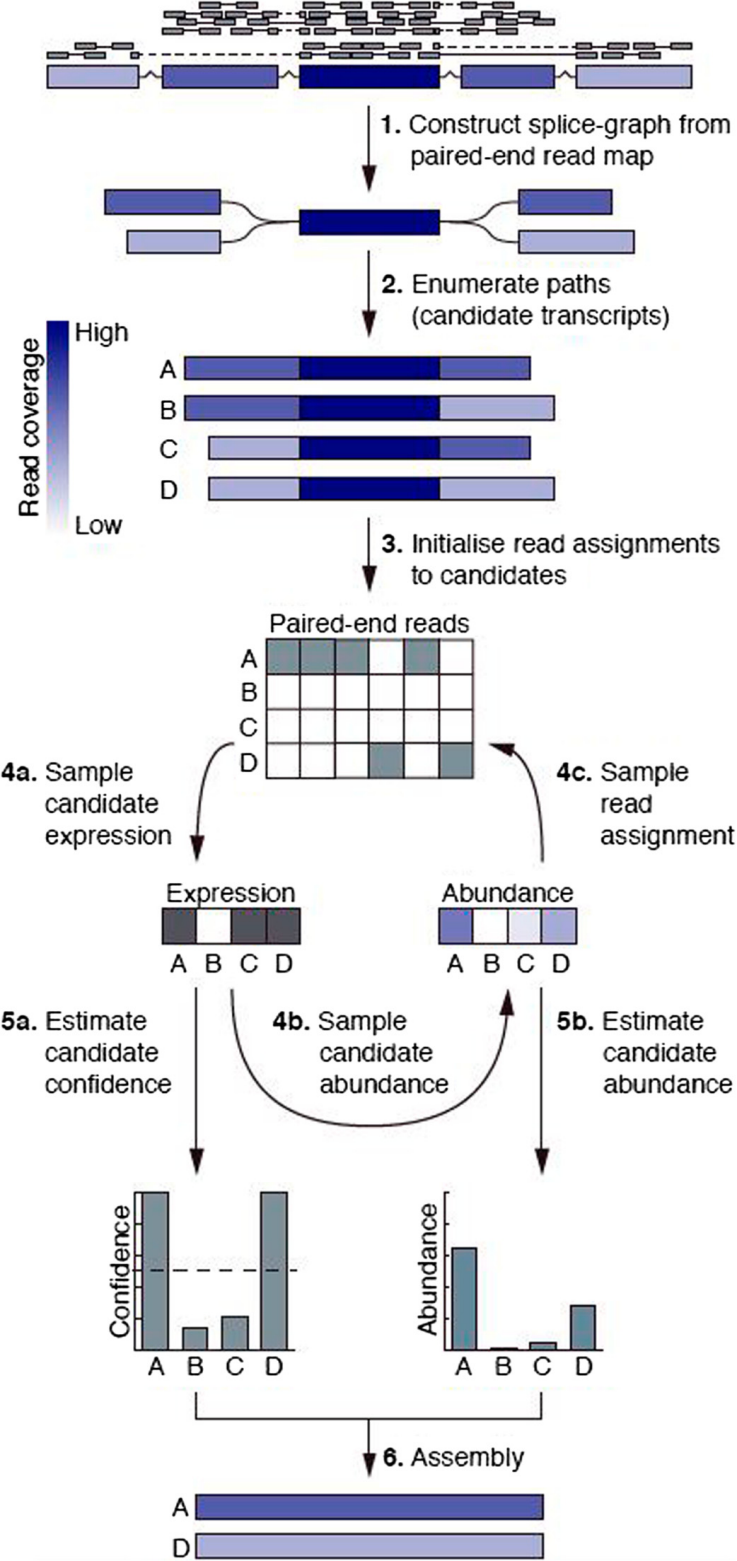
**Outline of the Bayesembler algorithm.** **(1)** A splice graph is first constructed from the RNA-seq data. **(2)** Transcript candidates are subsequently enumerated by exhaustively searching paths in the graph. **(3–5)** Gibbs sampling is then used to infer the posterior distribution over expressed candidates, their abundances and assignments of reads to candidates. **(3)** The sampler is initialised by randomly sampling a candidate assignment for each paired-end read and proceeds as follows. **(4a)** First, candidate expression values (i.e. binary values indicating whether the candidate has non-zero abundance) are sampled conditioned on an assignment of reads. **(4b)** Next, abundance values are sampled conditioned on the expressions and read assignments. **(4c)** Finally, read-to-candidate assignments are sampled conditioned on the transcript expression and abundance values, and the conditional probabilities of observing the reads given the candidates. **(5a,b)** The fraction of iterations a candidate transcript is expressed during sampling and its mean abundance level across expressed iterations are then used to estimate candidate confidence and abundance levels, respectively. **(6)** The final assembly is produced by selecting the transcript candidates with highest confidence.

The algorithm first constructs a set of splice graphs from a *TopHat2* [9] map of the RNA-seq reads using the graph construction routine in the *CEM* [5] assembly package (Figure 1(1)). Next, for each splice graph, a set of candidate transcripts is constructed by iteratively traversing paths in the graph and pruning the edges with lowest coverage until the total number of candidates does not exceed 100 (Figure 1(2)). Provided with a set of transcript candidates, we use Bayesian inference to determine the most likely combination of candidates and corresponding abundance levels.

Our inference method is built on a generative model of the RNA sequencing process. In the model, each candidate is associated with a binary random variable, which models whether the candidate is expressed (i.e. has non-zero abundance); this construct allows us to model our prior expectation of sparsity in the number of expressed candidates. Each expressed transcript is further associated with a real-valued random variable, which models its relative abundance. Finally, we assume that the binary variables share a Bernoulli prior distribution, which controls the number of expressed candidates, and further assume a symmetric Dirichlet prior distribution on the abundances of the expressed transcripts. Intuitively, this construct decouples the distribution of candidate expression from the distribution over abundance levels and hereby contrasts with most current approaches by not penalising lowly abundant transcripts. To specify a complete generative model of the RNA sequencing process, we assume that for each transcript a binary variable is first drawn from the Bernoulli distribution, followed by a draw of abundance values from the Dirichlet distribution for the expressed transcripts. For each paired-end read to be generated, a transcript is then drawn from the categorical distribution specified by the relative abundances, followed by sampling of a paired-end read from the transcript, essentially as described by Pachter [10].

A Gibbs sampling method was derived to infer the joint posterior distribution over expressed candidates, their abundances and assignments of reads to candidate transcripts, where the latter represents a latent variable in our model (Figure 1(3–5)). The Gibbs sampler is initialised by randomly sampling a candidate assignment for each read and proceeds as follows (Figure 1(3)). First, candidate expression values (i.e. binary values indicating whether a transcript has non-zero abundance) are sampled conditioned on an assignment of reads (Figure 1(4a)). Next, abundance values are sampled conditioned on the set of expressed transcripts and read assignments (Figure 1(4b)). Finally, each read is assigned to a candidate conditioned on the expression and abundance values of all candidates, and the probabilities of observing the read given each of the candidates (Figure 1(4c)). The number of iterations of these three steps needed to explore the posterior distribution sufficiently is then calculated as an affine function of the number of candidates. The main output of the algorithm is the fraction of iterations in which a candidate transcript is expressed (our confidence metric) and a posterior mean abundance estimate for each candidate (Figure 1(5a,b)). Candidates with a confidence above 0.5 and at least 12 expected paired-end read counts are included in the final assembly; the latter threshold is enforced to filter out putative transcript fragments (Figure 1(6)). The hyperparameter that controls the prior distribution over the number of expressed transcripts was estimated using a greedy minimum set cover method.

### Performance evaluation

The performance of our method was compared with state-of-the-art assemblers on both simulated data and data from the human K562 erythroleukaemia and H1 embryonic stem cell lines [11] as well as mouse dendritic cells [12]. We sought to compare against all assemblers that do not require a genome annotation and that are capable of handling paired-end data. Furthermore, we required that assemblers were stable and efficient enough to complete assembly on at least two of the employed datasets within a week of computation on a server with 40 CPUs. The *Cufflinks*, *IsoLasso*, *CEM* and *Traph* assemblers were selected based on these criteria. To retain a fair ground for comparison, a single set of optimal *Bayesembler* hyperparameters was estimated across datasets using partitions of the simulated, K562 and mouse dendritic cell data reserved for this purpose. Hence, the presented performance estimates for *Bayesembler* were obtained on hold-out partitions of these datasets together with the complete H1 dataset, which was reserved solely for testing purposes.

#### Simulated data

For the simulation study, a dataset of approximately 80 million paired-end strand-specific RNA-seq reads were simulated from the UCSC Known Genes annotation [13] using the *Flux Simulator* [14]. The main measures of performance were sensitivity, defined as the fraction of simulated transcripts that were assembled correctly, and precision, defined as the fraction of assembled transcripts found in the set of simulated transcripts.

**Figure 2:**
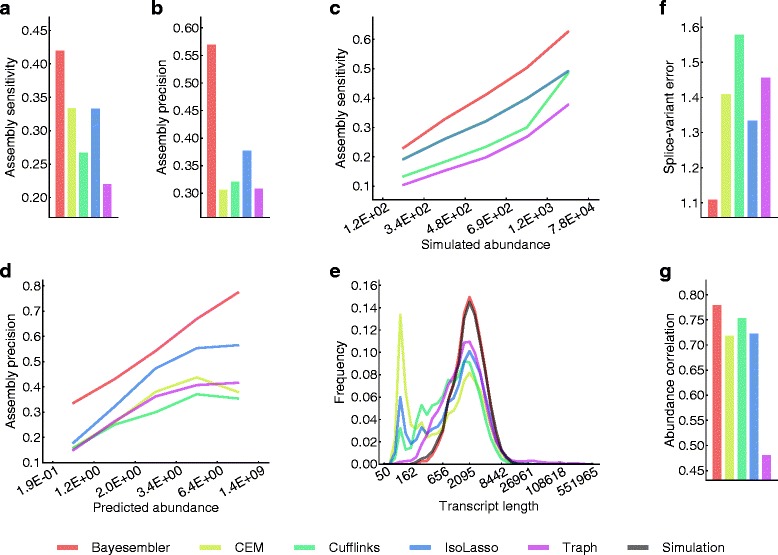
**Assembler performance on simulated RNA-seq data.** **(a)** Overall sensitivity, defined as the fraction of simulated transcripts that were predicted correctly. **(b)** Overall precision, defined as the fraction of predicted transcripts found in the set of simulated transcripts. **(c, d)** Sensitivity and precision as functions of simulated and predicted abundance, respectively. **(e)** Transcript length distributions of predicted and simulated transcripts (logarithmic binning). **(f)** Mean absolute difference between the number of predicted and the number of simulated splice variants for each predicted gene. **(g)** Spearman’s rank correlation between predicted and simulated abundances for transcripts predicted correctly by all five assemblers.

Our method exhibited both higher sensitivity and precision than all other methods (Figure 2a,b). More specifically, *Bayesembler* assembled 3,528 more correct transcripts, while producing 9,427 less incorrect ones on the data simulated from 40,496 annotated transcripts than the runner-up assembler, *IsoLasso*. Importantly, both sensitivity and precision remained higher for *Bayesembler* relative to the other assemblers independent of transcript abundance levels (Figure 2c,d). Moreover, the length distribution of transcripts predicted by *Bayesembler* resembled the length distribution of the simulated transcripts in contrast with the other assemblers, which tended to produce shorter transcripts (Figure 2e). Furthermore, *Bayesembler* was better at estimating the number of expressed splice variants for each predicted gene than the other assemblers, which may partially explain the observed improvements in assembly accuracy (Figure 2f, Figure S1 in Additional file 1). Finally, we assessed the accuracy of the transcript abundance estimates produced by the assemblers (Figure 2g, Figure S2 in Additional file 1). Here, the estimates produced by *Bayesembler* exhibited marginally better agreement with the simulated values compared with all other assemblers.

#### Real data

To assess performance on real data, the assemblers were tested on paired-end strand-specific RNA-seq data from two biological replicates from the K562 and H1 cell lines, and a single replicate of mouse dendritic cells. Importantly, these datasets represent different species, tissues, library construction protocols and sequencing depths. Of note, it was not possible to run the *Traph* assembler on the K562 and H1 data due to instability of the program. As there is no gold standard transcriptome reference for real data, we combined three complementary validation strategies to assess performance of the different assemblers.

**Figure 3:**
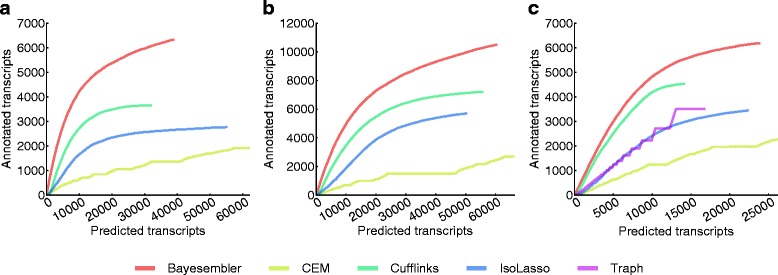
**Assembler performance estimates for real RNA-seq data using an annotation-based measure.** The number of assembled transcripts from **(a)** K562 (replicate 1), **(b)** H1 (replicate 1) and **(c)** mouse dendritic cells that were confirmed using the UCSC Known Genes annotation plotted against the corresponding number of predicted transcripts across a sequence of abundance thresholds (decreasing abundance threshold from left to right).

We first evaluated assembler performance by estimating the number of predicted transcripts that could be confirmed using the UCSC Known Genes annotation against the total number of predicted transcripts. To adjust for any abundance bias in the annotation and between assemblers, we calculated the number of confirmed transcript predictions across a sequence of thresholds on abundance and plotted it against the corresponding number of predicted transcripts. Hence, the final performance metric is a curve, where the height and slope represent sensitivity and precision, respectively. For both the K562 and H1 data, the *Bayesembler* curve extended higher and ascended more steeply for both replicates than any of the other assemblers thus indicating both better sensitivity and precision of our method (Figure 3a,b, Figure S3a,b in Additional file 1). Importantly, similar results were observed for data from mouse dendritic cells, which in turn suggests that the results are robust across species, tissues and library construction protocols (Figure 3c). Interestingly, we also observed that the transcript lengths produced by *Bayesembler* were closer to the length distribution of annotated transcripts than the other assemblers, which again tended to produce shorter transcripts (Figure S4a–e in Additional file 1).

**Figure 4:**
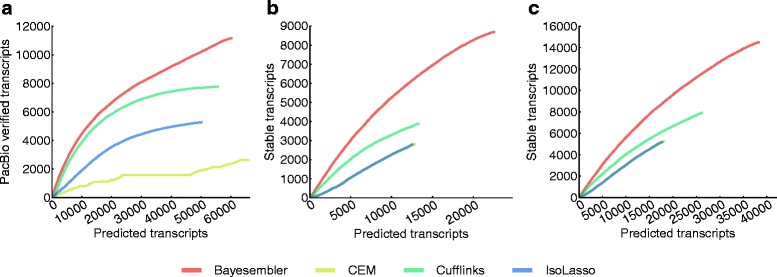
**Assembler performance estimates for real RNA-seq data using PacBio and replicate stability-based measures.** **(a)** The number of assembled transcripts from H1 (replicate 1) that were verified by a PacBio read against the corresponding number of predicted transcripts across a sequence of abundance thresholds (decreasing abundance threshold from left to right). The number of stable transcripts (i.e. predicted multi-exonic transcripts that were identical between replicates) from **(b)** K562 and **(c)** H1 plotted against the corresponding number of predicted transcripts across a sequence of thresholds on transcript rank, where the ranks were obtained by sorting transcripts in each replicate in decreasing order of abundance (decreasing rank threshold from left to right).

Next, we evaluated both replicate assemblies of the H1 transcriptome using data from a recent study by Au *et al.* [15]. In this study, RNA from the same cell line was sequenced on the Pacific Biosciences (PacBio) platform (Pacific Biosciences, California, USA), which produces significantly longer reads than standard second-generation sequencing platforms at the expense of lower throughput. As sampling bias implies that lowly abundant transcripts have a lower probability of being verified by a PacBio read, we again used the curve-based metric introduced above to adjust for transcript abundance bias between assemblers. Hence, we computed the number of transcript predictions that could be verified by a PacBio read against the corresponding full number of predictions across a sequence of thresholds on abundance (Figure 4a, Figure S5 in Additional file 1). In agreement with the annotation-based results, *Bayesembler* found more verified transcripts both in absolute numbers and relative to the total number of predicted transcripts for each threshold, thus demonstrating better sensitivity and precision, respectively.

Finally, we leveraged the large degree of overlap expected between the transcriptomes of biological replicates to evaluate the different assemblers. We defined stable transcripts as multi-exonic transcripts that were present in both replicate assemblies. Again, we used a curve-based metric to correct for abundance bias between assemblers as lowly abundant transcripts are expected to be less stable. More specifically, for each pair of K562 and H1 replicates, assembled transcripts were ranked according to abundance for each of the replicates and the number of stable transcripts above a certain rank was plotted against the corresponding number of predicted transcripts to produce a curve with a point for each rank (Figure 4b,c). As seen in the figure, transcripts predicted by *Bayesembler* were generally more stable across replicates than those of any of the other assemblers, suggesting improved performance. We further used the replicate assemblies to evaluate the impact of noise on transcript abundance estimation by comparing the estimates across replicates (Figures S6 and S7 in Additional file 1). Only minor differences in replicate abundance correlations were observed between the different assemblers, with *Bayesembler* performing best for the K562 data and *Cufflinks* performing best for the H1 data.

## Discussion

We have devised a new probabilistic approach to transcriptome assembly. Our primary result is the derivation of a Bayesian model of the RNA sequencing process, which uses a novel prior distribution over transcript abundances to model the number of expressed transcripts for each gene. The model thus provides a statistically consistent way of combining *a priori* knowledge about sparsity in the number of expressed transcripts with information from the sequencing data. Importantly, in the model, only the read distribution along transcript sequences – and not transcript abundance levels – influences which transcripts are inferred as expressed. This contrasts with most current assemblers, which use abundance – either implicitly through regularisation or explicitly by truncating assemblies at an abundance cut-off – as a proxy for transcript confidence. This is despite the fact that abundance contains only limited information about whether a transcript is expressed in a sample.

The main advantage of our fully probabilistic approach is the ability to quantify our degree of confidence in a transcript given the data. To our knowledge, all previously published assemblers output assembly point estimates and thus provide no means of prioritising newly discovered variants for downstream validation. In contrast, *Bayesembler* provides both a confidence and an abundance estimate for each transcript. To evaluate our confidence metric, we used a simple threshold on confidence – and expected read count – to determine the most likely assembly from one simulated and five real datasets and compared our performance with those of state-of-the-art assemblers. Measuring the accuracy of transcriptome assembly algorithms without extensive experimental validation is difficult. More specifically, simulated data provides a known ground truth at the cost of less realistic splicing and noise patterns, whereas real-data benchmarks suffer from the lack of a ground truth reference. We therefore used a combination of simulations and three complementary metrics applied across different real datasets to gauge performance.

For simulated data, *Bayesembler* markedly outperformed all other assemblers on both sensitivity and precision, with *IsoLasso* coming in as runner-up. Interestingly, *Bayesembler* was markedly better at estimating the number of expressed transcripts for each gene, thus lending confidence to our sparsity model. In contrast, *Cufflinks* markedly underestimated the number of transcripts for many genes, suggesting that its strong sparsity objective negatively affects both sensitivity and precision. Real-data benchmarking was performed by comparing assemblies with both transcript annotations, PacBio long-read sequencing data and independent assemblies made for biological replicates to adjust for biases in the individual verification methods. As the latter two reference sets (and likely also annotations) all favour highly abundant transcripts, we used a novel curve-based metric to take into account any possible abundance bias between assemblers. *Bayesembler* consistently outperformed all other assemblers, with *Cufflinks* coming in as runner-up for all metrics for all datasets. Clearly, the validity of the real-data performance metrics is strengthened because the same ranking of assembler performance was observed across metrics and datasets. The performance ranking of *IsoLasso* and *Cufflinks* was inconsistent between the benchmarks for simulated and real data. We speculate that annotation-based simulations may give rise to genes with more complex splicing patterns (i.e. more variants per gene), which may have impaired the performance of *Cufflinks*. Abundance estimation accuracy was also investigated, with *Bayesembler* and *Cufflinks* performing better than *CEM* and *IsoLasso*, but the results did not allow for performance ranking of the former two assemblers.

Importantly, the gain in accuracy provided by *Bayesembler* does not come at the cost of increased computation time. Indeed, our program took approximately 5.5 hours on 16 CPU cores to assemble the deepest benchmark dataset (approximately 125 million paired-end reads) with a maximum memory footprint of 1.7 GB. This is both faster and more memory efficient than the widely used *Cufflinks* assembler.

Looking ahead, we believe that our method will also benefit fields outside of reference-based transcriptome assembly. First, we note that our probabilistic inference method is also directly applicable to *de novo* assembled splice graphs and could easily be implemented as a post-processing routine in packages like *Trinity* [12] and *Oases* [16]. Second, the ability to consistently average quantities that depend on the transcript structure provides an additional advantage of our probabilistic approach. Indeed, a recent study highlighted the influence of transcript structure on gene expression estimates [17] and we speculate that gene expression estimates averaged across assemblies may improve the accuracy of differential expression tests.

## Conclusions

RNA-seq is rapidly becoming the *de facto* standard method for expression analysis. However, despite vast amounts of available data, the reconstruction of complete transcripts from such data remains a fundamental challenge in computational biology.

We present here *Bayesembler*, a statistical approach to transcriptome assembly based on a novel Bayesian model and inference method. Using our approach, we observe a marked and consistent improvement both in assembly sensitivity and precision over state-of-the-art assemblers as judged using several independent robust measures applied across several datasets. Moreover, the use of a fully probabilistic approach allows us to provide a confidence (and an abundance) estimate for each transcript, which we expect will aid in prioritising newly discovered transcripts for experimental validation.

## Materials and methods

Our transcriptome assembly algorithm proceeds by first constructing a set of splice graphs [1] (i.e. a directed acyclic graph where vertices represent exons and edges represent exon–exon junctions) from an alignment of RNA-seq reads. Next, for each splice graph, transcript candidates are enumerated by exhaustively searching paths in the graph. Bayesian inference is then used to identify the most probable transcript candidates and associated abundance levels given the reads using a generative model of the RNA sequencing process.

### Splice graph construction and generation of transcript candidates

Provided with a *TopHat2* [9] alignment of RNA-seq reads, putative PCR duplicates and multimapping reads are first removed. Furthermore, only reads mapping in a proper paired-end fashion are retained. For strand-specific data, the originating strand is inferred for each paired-end read and splice graphs are constructed separately for each strand using the *processsam* utility from the *CEM* (v0.9.1) [5] transcriptome assembly package with the -d <strand> option. For unstranded data, the originating strand is inferred from splice site information by running *processsam* with the -d. option. All graphs for which a strand cannot be inferred are discarded by the assembler. For both stranded and unstranded data, *processsam* is run with a minimum gap length between genes of one and a minimum number of two reads per gene (-g 1 -c 2).

A set of candidate transcripts is then generated for each splice graph using the following iterative procedure. The algorithm is first initialised with an edge-coverage threshold of one read. For each splice graph, all source-to-sink paths are then enumerated using a depth-first search until the search is complete or the number of candidates exceeds 100. In the latter case, the edge-coverage threshold is incremented by one and all edges with a coverage below the threshold removed from the graph; the graph-search and edge-pruning steps are iterated until the path search has been completed without exceeding the threshold or all edges have been removed. To model the presence of pre-mRNA in the sample, a candidate with a single exon spanning the entire genomic interval of the splice graph is included. Next, to leverage paired-end information, only candidates with paired-end read coverage across all splice junctions are retained. Finally, to improve detection of pre-mRNA material, candidates from overlapping splice graphs are combined and the resulting candidate sets used as bases for the probabilistic inference method.

### A generative model of the RNA-sequencing process

We will use *sequencing fragment* to denote a read pair. The overall process of generating a set *F* of *n* sequencing fragments given a set *S* of *m* candidate transcripts proceeds as follows. First, a set of relative transcript abundances taking values in [0,1] is drawn from a prior distribution. The inclusion of point-zero abundances in the sample space reflects prior knowledge that only a subset of the candidates are likely to be expressed. For each fragment to be generated, a transcript candidate is then sampled conditioned on the abundance values followed by sampling the fragment’s two sequencing reads conditioned on the selected candidate.

To define a prior distribution that allows for point-zero transcript abundances, let first **z** be a vector of *m* independent random variables drawn from the Bernoulli distribution with parameter *π* such that:

$$\mathbb{P}(\mathbf{z}|\pi) = \pi^{b_{\mathbf{z}}}\left(1-\pi\right)^{m-b_{\mathbf{z}}} K_{\mathbf{z}_{0}} $$

where:

$$\begin{aligned} b_{\mathbf{z}}&=\sum_{j=1}^{m} z_{j}\quad \text{and} \quad K_{\mathbf{z}_{0}}&=\frac{1}{1-\left(1-\pi\right)^{m}} \end{aligned} $$

(Supplementary methods 1 in Additional file 1). Then, let *z*_*j*_=0 and *z*_*j*_=1 indicate that transcript *s*_*j*_ has point-zero abundance (i.e. *s*_*j*_ is not expressed) and positive abundance (i.e. *s*_*j*_ is expressed), respectively. Next, let **e**^+^ be a vector containing the relative abundance levels for the expressed transcripts such that:

$$|\mathbf{e}^{+} |=b_{\mathbf{z}} \quad \text{and} \quad \sum_{k=1}^{b_{\mathbf{z}}}e_{k}^{+}=1, $$

and let the values be symmetrically Dirichlet distributed such that:

$$\mathbb{P}(\mathbf{e}^{+}|\mathbf{z},\gamma)=\frac{\Gamma\left(b_{\mathbf{z}} \gamma\right)}{\Gamma\left(\gamma\right)^{b_{\mathbf{z}}}}\prod_{k=1}^{b_{\mathbf{z}}}\left(e_{k}^{+}\right)^{\gamma-1} $$

where *Γ* is the gamma function. Finally, define **e** to be the vector of transcript abundances taking values in [0,1] and let it be completely specified by **z** and **e**^+^ such that *e*_*j*_=0 when *z*_*j*_=0 and $e_{j}=e_{k}^{+}$ when *z*_*j*_=1, where:

$$k=\sum_{l=1}^{j}z_{l} $$

Then, to generate a set of abundance values, *m* binary values are first drawn from the Bernoulli distribution conditioned on the parameter *π* followed by sampling of **e**^+^ from the *b*_**z**_-dimensional symmetric Dirichlet distribution. Conditioned on the set of abundances, each fragment *f*∈*F* is then generated by first sampling a transcript index *t* from the categorical distribution $\mathbb {P}(t|\mathbf {e})$ defined by **e** (i.e. $\mathbb {P}(t|\mathbf {e})=e_{t}$). Finally, conditioned on the candidate index, the fragment sequences are sampled from $\mathbb {P}(f|t,q,S,\mu,\sigma)$, where *q*∈*Q* represent the observed quality scores for fragment *f*, and *μ* and *σ* denote the mean and standard deviation of the fragment length distribution, respectively, essentially as proposed by Pachter [10].

The joint distribution over a set of *n* fragments *F*, transcript indices **t** and transcript abundances **e** conditioned on the set of transcript candidates, quality scores and hyperparameters then factorises as

$$\begin{array}{*{20}l} &\mathbb{P}(F,\mathbf{t},\mathbf{e}|Q,S,\mu,\sigma,\pi,\gamma)=\\ &\mathbb{P}(\mathbf{e}|\pi,\gamma) \prod_{i=1}^{n}\mathbb{P}\left(\,f_{i}|t_{i},q_{i},S,\mu,\sigma\right)\mathbb{P}(t_{i}|\mathbf{e}) \end{array} $$

with

$$\mathbb{P}(\mathbf{e}|\pi,\gamma) = \mathbb{P}\left(\mathbf{e}^{+},\mathbf{z}|\pi,\gamma\right)=\mathbb{P}\left(\mathbf{e}^{+}|\mathbf{z},\gamma\right)\mathbb{P}(\mathbf{z}|\pi) $$

**Figure 5:**
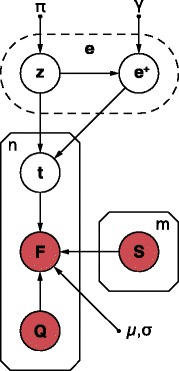
**Graphical model of the joint probability distribution.** The arrows indicate dependencies between random variables. Observed and unobserved random variables are coloured in *red* and *white*, respectively. Small filled circles indicate hyperparameters. The variables and hyperparameters are explained in the Materials and methods section and in Supplementary methods 1 in Additional file 1.

The corresponding graphical model is shown in Figure 5. Further details of the model derivation are provided in Supplementary methods 1 in Additional file 1.

### Approximate inference using Gibbs sampling

To apply the model to transcriptome assembly, we need to infer the posterior distribution over abundance levels **e**. This will in turn provide information on both whether a given transcript candidate *s*_*j*_ is expressed (i.e. when *e*_*j*_>0) and its abundance value if expressed. We treat the vector of transcript indices **t** as a nuisance variable to be inferred to make inference tractable, and $\mathbb {P}(\mathbf {t},\mathbf {e}|F,Q,S,\mu,\sigma,\pi,\gamma)$ thus becomes the target posterior distribution. Samples from this joint distribution are obtained by iteratively drawing samples from $\mathbb {P}(\mathbf {e}|\mathbf {t},\pi,\gamma)$ and $\mathbb {P}(\mathbf {t}|\mathbf {e},F,Q,S,\mu,\sigma)$. To do this, let **c** denote the vector of occurrences of each transcript index in **t** (i.e. |**c**|=*m* and $\sum _{j=1}^{m}c_{j} =n$). It follows from the model definition that **c** is a sufficient statistic for **t** with respect to the posterior distribution of **e**. From this and our definition of the prior distribution over abundances, it follows that:

$$\mathbb{P}\left(\mathbf{e}|\mathbf{t},\pi,\gamma\right)=\mathbb{P}\left(\mathbf{e}^{+}|\mathbf{z},\mathbf{c},\gamma\right)\mathbb{P}\left(\mathbf{z}|\mathbf{c},\pi,\gamma\right) $$

A sample from $\mathbb {P}(\mathbf {e}|\mathbf {t},\pi,\gamma)$ can then be obtained by first drawing the number of expressed transcripts *b*_**z**_ from:

$${} \mathbb{P}(b_{\mathbf{z}}|\mathbf{c},\pi,\gamma) = \frac{\left(\!\!\begin{array}{c}|J^{0}|\\{b_{\mathbf{z}}-|J^{+}|}\end{array}\!\!\right)\frac{\Gamma(b_{\mathbf{z}}\gamma)}{\Gamma(n+b_{\mathbf{z}}\gamma)}\pi^{b_{\mathbf{z}}}(1-\pi)^{m-b_{\mathbf{z}}}}{\sum_{b=|J^{+}|}^{m} \left(\!\!\begin{array}{c}|J^{0}|\\{b-|J^{+}|}\end{array}\!\!\right)\frac{\Gamma(b\gamma)}{\Gamma(n+b\gamma)}\pi^{b}(1-\pi)^{m-b}} $$

where *J*^+^ is the subset of transcript indices *j* for which *c*_*j*_>0, *J*^0^ is the subset of indices for which *c*_*j*_=0 and *b*_**z**_ is constrained such that *J*^+^≤*b*_**z**_≤*m*. Conditioned on *b*_**z**_, a binary vector **z** is first generated such that *z*_*j*_=1 for all *j*∈*J*^+^. The remaining transcripts are then allocated by sampling *b*_**z**_−|*J*^+^| transcript indices *H* uniformly from *J*^0^ and setting *z*_*h*_=1 for all *h*∈*H*. Equivalent to the definition of **e**^+^, let **c**^+^ denote the vector of occurrences *c*_*j*_ for which *z*_*j*_=1. The abundance levels **e**^+^ are then sampled from:

$$\mathbb{P}\left(\mathbf{e^{+}}|\mathbf{z},\mathbf{c},\gamma\right) = \frac{\Gamma(n+b_{\mathbf{z}}\gamma)}{\prod_{k=1}^{b_{\mathbf{z}}}\Gamma\left(c_{k}^{+} + \gamma\right)} \prod_{k=1}^{b_{\mathbf{z}}} \left(e_{k}^{+}\right)^{c_{k}^{+}+\gamma-1} $$

Finally, it follows from conditional independence of the elements in **t** given **e** that a sample from $\mathbb {P}(\mathbf {t}|\mathbf {e},F,Q,S,\mu,\sigma)$ can be obtained by sampling the individual elements of **t** from:

$$\mathbb{P}(t|\mathbf{e},f,q,S,\mu,\sigma)=\frac{\mathbb{P}\left(\,f|t,q,S,\mu,\sigma\right)\mathbb{P}(t|\mathbf{e})}{\sum_{j=1}^{m}\mathbb{P}\left(\,f|j,q,S,\mu,\sigma\right)\mathbb{P}\left(j|\mathbf{e}\right)} $$

The sampler is initialised by randomly sampling a fragment assignment **t**. A detailed derivation of the Gibbs sampling updates is provided in Supplementary methods 2 in Additional file 1. To estimate *π*, the minimum number of transcript candidates required to explain all paired-end reads *m*_min_ is obtained using a greedy method and *π* estimated using:

$$\pi=\frac{m_{\text{min}}}{m} $$

(Supplementary methods 3 in Additional file 1). The fragment lengths of all paired-end reads mapping uniquely to transcripts at least 2500 nt long from single-transcript graphs are used to estimate the parameters of the Gaussian distribution used to model fragment lengths. To minimise the influence of outliers, we use the *median* and *median absolute deviance* as estimators of *μ* and *σ*, respectively (Supplementary methods 3 in Additional file 1). Finally, an uninformative choice of *γ*=1 corresponding to the uniform symmetric Dirichlet distribution is used for the prior over relative abundances. The number of burn-in iterations required for each graph is calculated as 60×*m*+1000, where *m* is the number of candidates; the number of subsequent iterations (i.e. the sample size) is set to 10 times the number of burn-in iterations.

The samples from $\mathbb {P}(\mathbf {e}|\mathbf {t},\pi,\gamma)$ are subsequently used to estimate both a confidence and a mean abundance estimate for each transcript candidate, where the confidence estimate is calculated as the fraction of iterations in which a candidate is expressed. The posterior mean abundance estimate is calculated as the average of the sampled abundances after they have been normalised to the effective transcript length and total library size (Supplementary methods 2 in Additional file 1). The final assembly is produced by selecting all transcript candidates with a confidence above 0.5 and excluding transcripts with an expected paired-end read count below 12; the latter threshold was implemented to filter out putative transcript fragments.

### Implementation and availability

The inference algorithm is implemented in C++ as a complete transcriptome assembly package under the name *Bayesembler* and supports multi-threading. The program is freely available under the MIT licence [18]. The algorithm takes a *TopHat2* [9] map as input and outputs an assembly in GTF format and is thus compatible with downstream analysis tools like *CuffDiff2* [17].

### Benchmarking

The *Flux Simulator* [14] (v1.2) was used to simulate approximately 80 million strand-specific paired-end reads from the human UCSC Known Genes annotation [13] (hg19 version downloaded 10 May 2013) with read lengths of 100 nt and a mean fragment length of 249 nt. Reads were simulated without variation in transcription start and end sites using the *fragmentation-first* protocol, where fragmentation is performed prior to reverse transcription with all other parameters set to their default values. Paired-end strand-specific RNA-seq data from two biological replicates of the K562 and H1 cell lines [11], and a single replicate of mouse dendritic cells [12] together with PacBio reads [15] from the H1 cell line were downloaded from the NCBI Short Read Archive and the Gene Expression Omnibus database (Table 1). Simulated and real RNA-seq reads were mapped to either the hg19 or mm10 reference genomes using *Tophat2* [9] (v2.0.8, default settings) together with *Bowtie2* [19] (v2.1.0). The simulated, K562 and mouse data were divided into separate *validation* and *test* sets each consisting of data from half of the chromosomes. The H1 data were reserved for testing purposes only. The validation sets were used for estimating the maximum number of candidates, how the number of Gibbs iterations should scale with the number of candidates as well as the transcript confidence and expected count thresholds. Hence, the test datasets were used exclusively to produce the benchmarks presented in this paper.

**Table 1:** **Datasets used in the benchmark**

**Sample**	**Platform**	**Approximate depth**	**Accession**	**Reference**
Human K562 cells(replicate 1)	Illumina	125 million	[SRX110318] ^*a*^	[11]
Human K562 cells(replicate 2)	Illumina	88 million	[SRX110318] ^*a*^	[11]
Human H1 cells(replicate 1)	Illumina	41 million	[SRX082572] ^*a*^	[11]
Human H1 cells(replicate 2)	Illumina	37 million	[SRX082572] ^*a*^	[11]
Human H1 cells	PacBio	8 million	[GSE51861] ^*b*^	[15]
Mouse dendriticcells	Illumina	53 million	[SRX062280] ^*a*^	[12]

^a^NCBI Sequence Read Archive.

^b^NCBI Gene Expression Omnibus.

The *Cufflinks* [2] (v2.1.1), *IsoLasso* [3] (v2.6), *CEM* [5] (v0.9.1) and *Traph* [8] (v0.7) assemblers were used in the performance evaluation. All assemblers were run with default parameters except that bias estimation was enabled for *Cufflinks*, *IsoLasso* and *CEM*, and *Cufflinks* was also run with multimap correction. In addition, the fragment length mean and standard deviation estimates used by *Bayesembler* were provided as input to *IsoLasso* and *CEM*. *Bayesembler* (v1.1.1) was used in the performance evaluation.

The main benchmark criteria for the simulated data were *sensitivity*, defined as the fraction of simulated transcripts that were predicted correctly, and *precision*, defined as the fraction of predicted transcripts that were found in the simulation set. Here, a transcript match was defined as a complete intron-chain match between an assembled transcript and a simulated transcript (i.e. identical intron coordinates between the two transcripts). Single exon transcripts were considered matches if they were contained in and covered at least 75% of a simulated single-exon transcript. As neither *CEM*, *IsoLasso* nor *Traph* provides full support for strand-specific data, transcripts were not matched by strand to provide a conservative estimate of our performance relative to these assemblers. Of note, the abundance estimates of all other assemblers were renormalised to the total library size to allow for visual comparison with the *Bayesembler* estimates to produce Figure 2d and Figures S2, S6 and S7 in Additional file 1. Spearman’s rank correlation coefficient was used to assess the accuracy of the abundance estimates for each assembler; this metric was selected to provide robustness to the scale of abundances and outliers. To adjust for assembly size, only abundance values of transcripts assembled correctly by all assemblers (i.e. the intersection between assemblies and the simulation set) were used to compute the correlations.

Performance on the five real datasets was first assessed by plotting the number of assembled transcripts that could be confirmed using the UCSC Known Genes annotations (hg19 version downloaded 10 May 2013; mm10 version downloaded 1 October 2013) against the corresponding number of predicted transcripts in each assembly. These estimates were computed across a sequence of lower-bound thresholds on abundance to adjust for any abundance bias in the confirmation method using the same transcript matching criteria as defined above for the simulated transcripts. The same metric was also used for further evaluation of the assemblers’ performance on the H1 datasets using PacBio reads for confirmation. Finally, performance was also estimated by assessing the overlap between replicate assemblies from the K562 and H1 datasets. To do this, we defined stable transcripts as multi-exonic transcripts that were present in both replicate assemblies. Assembled transcripts were ranked according to abundance for each of the two replicates, and the number of stable transcripts was plotted against the corresponding number of predicted transcripts above a certain rank, thus producing a curve with a point for each rank. Finally, Spearman’s rank correlation coefficient was computed for the predicted abundances of transcripts assembled for both replicates. Only the intersection between stable transcripts of the different assemblers was used to compute the correlations to adjust for assembly size.

## Additional file

Additional file 1
**Supplementary information.** Supplementary figures S1–S7 and Supplementary methods 1–3.

## Abbreviations

PacBio: Pacific Biosciences
PCR: polymerase chain reaction
RNA-seq: RNA sequencing
