# Supplementary material for: Bayesian transcriptome assembly
Source: Genome Biol. 2014 Oct 31;15(10):501. doi: 10.1186/s13059-014-0501-4 (PMC4397945; doi:10.1186/s13059-014-0501-4)
Supplement: Additional file 1 — Supplementary information. Supplementary figures S1–S7 and Supplementary methods 1–3. [file 13059_2014_501_MOESM1_ESM.pdf]

Bayesian transcriptome assembly

Supplementary information

Lasse Maretty[*1], Jonas Andreas Sibbesen[*1] and Anders Krogh[1]

[*]Equal contributors.
[1]The Bioinformatics Centre, Department of Biology and Biotech Research and Innovation Centre (BRIC), University of Copenhagen, Ole Maaløes Vej 5, 2200 Copenhagen, Denmark.
Correspondence should be addressed to A.K. (krogh@binf.ku.dk).

Contents

Supplementary figures **2**
Figure S1: Difference in predicted and simulated number of variants per gene. . . 2
Figure S2: Abundance estimation accuracy on simulated data 3
Figure S3: Assembler performance estimates on K562 and H1 replicate 2 - annotation-based measure 4
Figure S4: Length distribution of assembled transcripts 5
Figure S5: Assembler performance estimates on H1 replicate 2 - PacBio-based measure 6
Figure S6: Inter-replicate correspondence of abundance estimates on K562 7
Figure S7: Inter-replicate correspondence of abundance estimates on H1 8

Supplementary methods **9**
1 A generative model of the RNA-sequencing process 9
1.1 A prior distribution over transcript candidate abundances 9
1.2 A generative model of the sequencing process of a transcript 10
1.3 The joint probability distribution 11
2 Approximate inference using Gibbs sampling 12
2.1 Sampling abundance levels 12
2.2 Sampling fragment assignments 14
2.3 Abundance normalisation 15
3 Hyperparameter estimation 16
3.1 Fragment length distribution estimation 16
3.2 Sparsity estimation 16

Supplementary references **17**

Supplementary figures

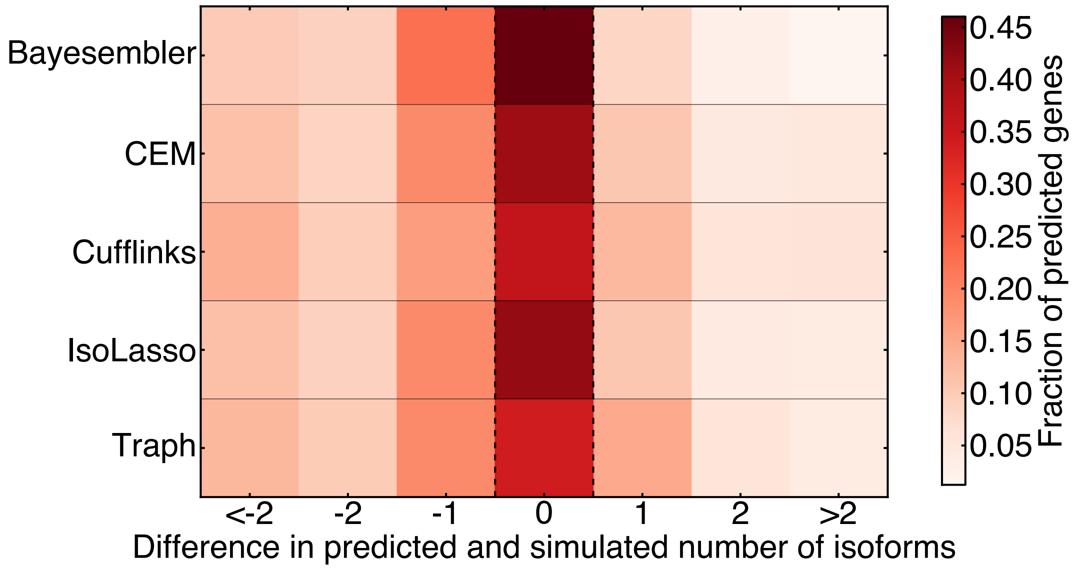

Figure S1: Difference in predicted and simulated number of variants per gene. RNA-sequencing of transcripts from the UCSC Known Genes annotation [1] was simulated using the *Flux Simulator* [2]. The figure shows the distribution over the differences in the number of predicted transcripts and the number of simulated transcripts for each predicted gene.

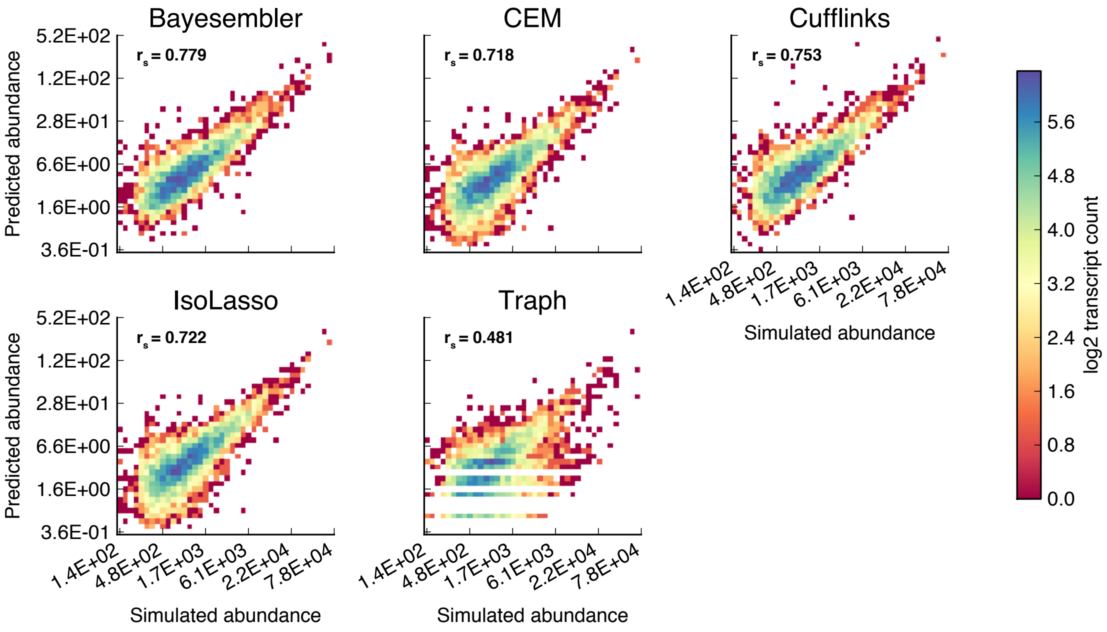

Figure S2: Abundance estimation accuracy on simulated data. RNA-sequencing of transcripts from the UCSC Known Genes annotation [1] was simulated using the *Flux Simulator* [2]. The figures show predicted abundance estimates against the corresponding simulated abundances for transcripts that were predicted correctly by all five assemblers. r_s denotes the Spearman's rank correlation coefficient.

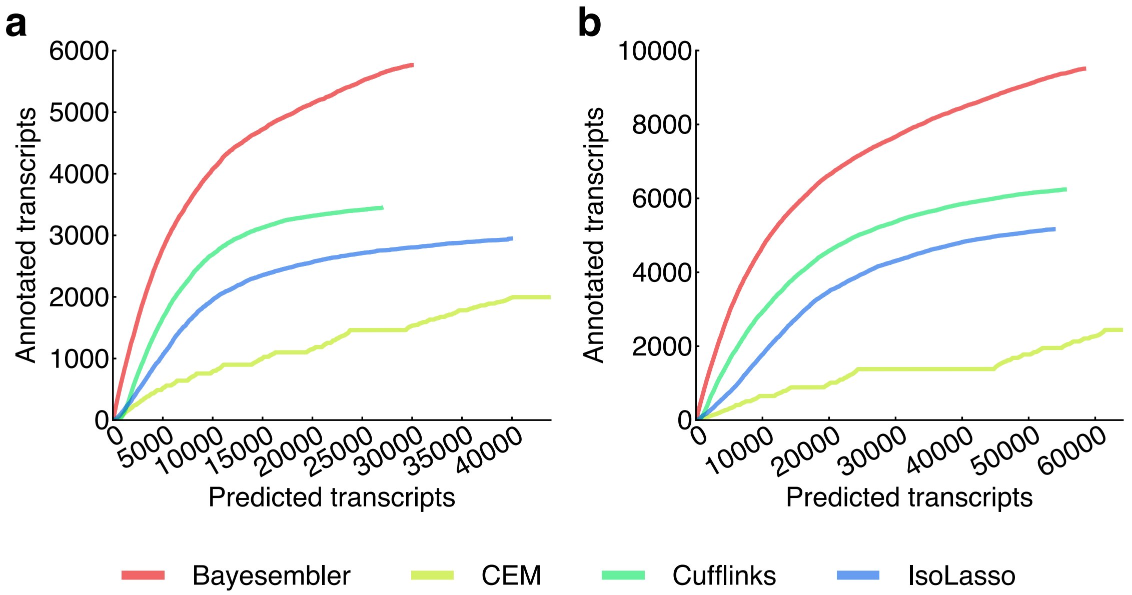

Figure S3: Assembler performance estimates on K562 and H1 replicate 2 - annotation-based measure. The figures show the number of assembled transcripts from K562 (replicate 2) [3] (**a**) and H1 (replicate 2) [3] (**b**) that were confirmed using the UCSC Known Genes annotation [1] against the corresponding number of predicted transcripts across a sequence of abundance thresholds (decreasing abundance threshold from left to right).

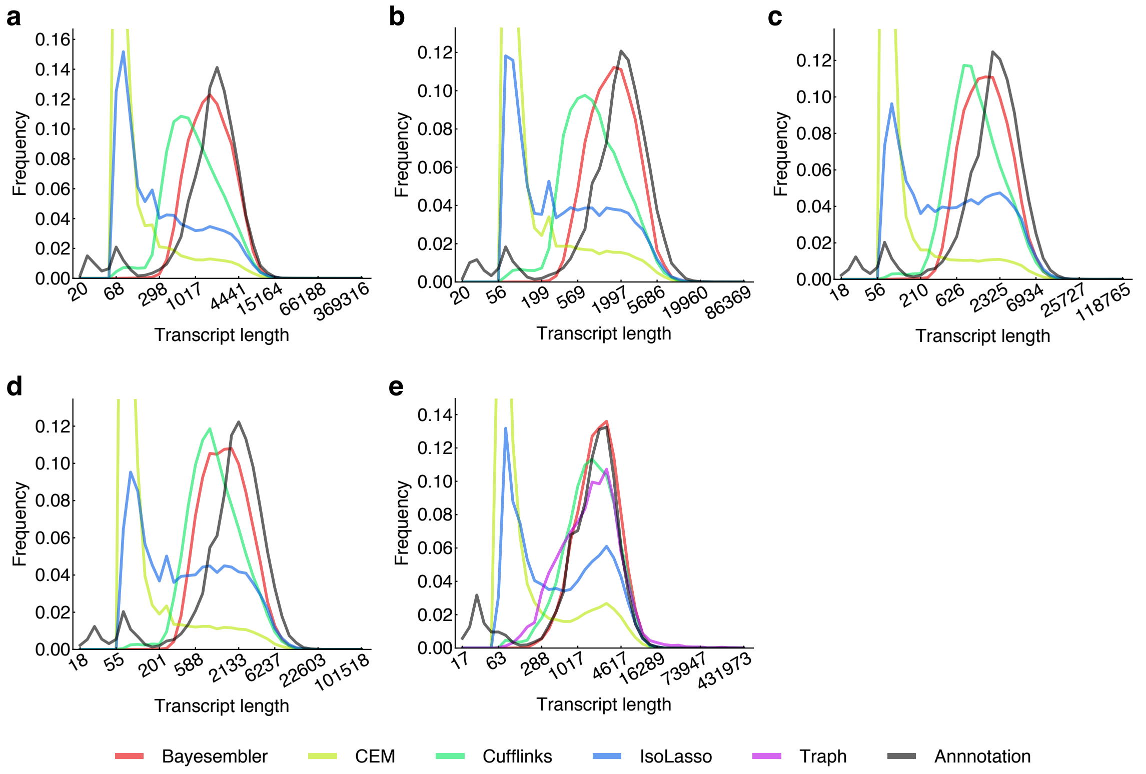

Figure S4: Length distribution of assembled transcripts. The figures show the length distribution of the assembled transcripts from K562 (replicate 1 and 2) [3] (**a**,**b**), H1 (replicate 1 and 2) [3] (**c**,**d**) and mouse dendritic cells [4] (**e**) together with the length distribution of the UCSC Known Genes annotation [1] (logarithmic binning).

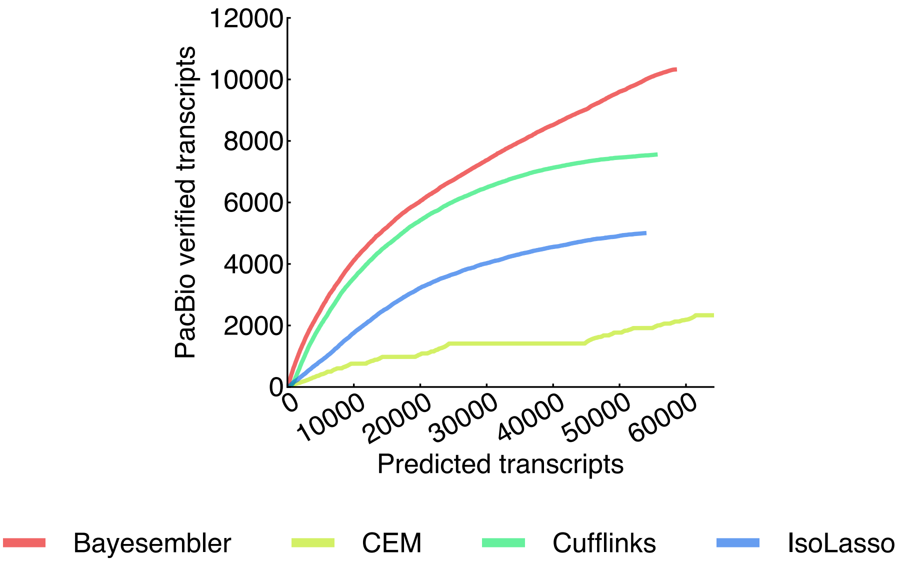

Figure S5: Assembler performance estimates on H1 replicate 2 - PacBio-based measure. The figure show the number of assembled transcripts from H1 (replicate 2) [3] that were verified by a PacBio read [5] against the corresponding number of predicted transcripts across a sequence of abundance thresholds (decreasing abundance threshold from left to right).

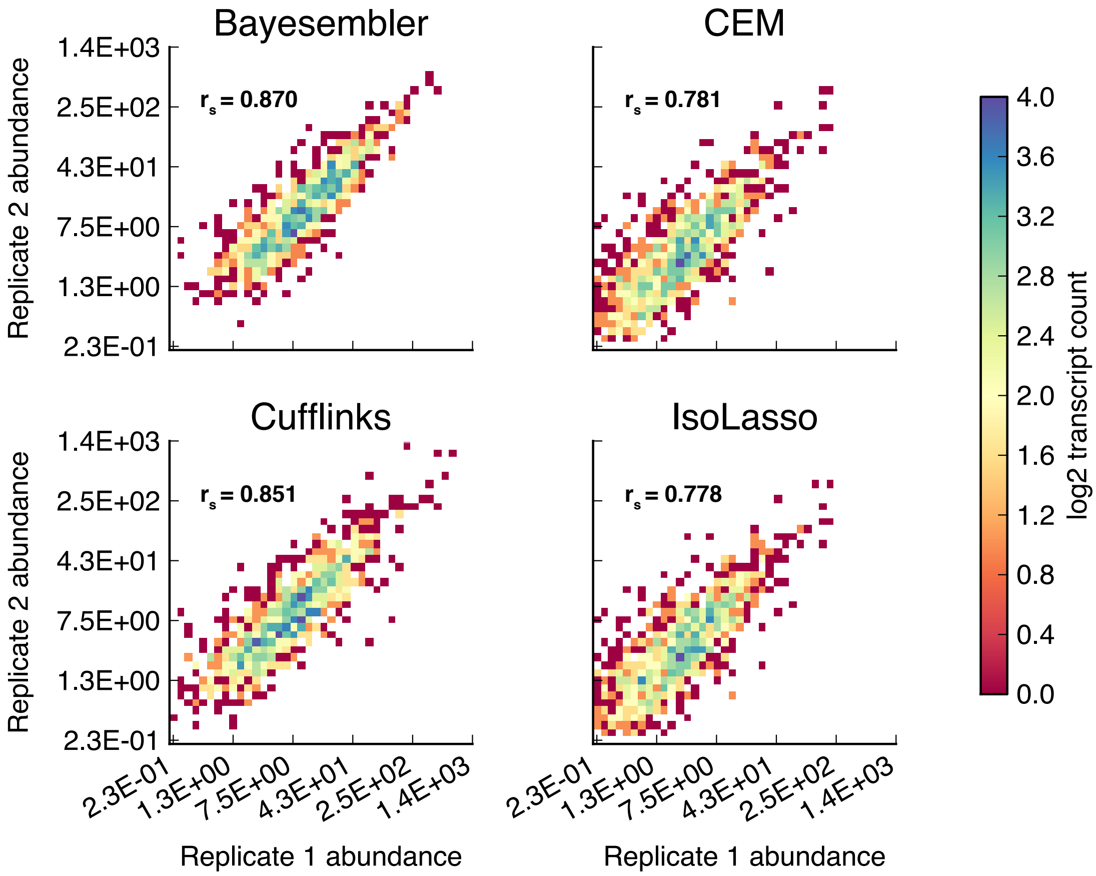

Figure S6: Inter-replicate correspondence of abundance estimates on K562. The figures show the predicted abundances of transcripts assembled in both K562 replicates [3] by all assemblers. r_s denotes the Spearman's rank correlation coefficient.

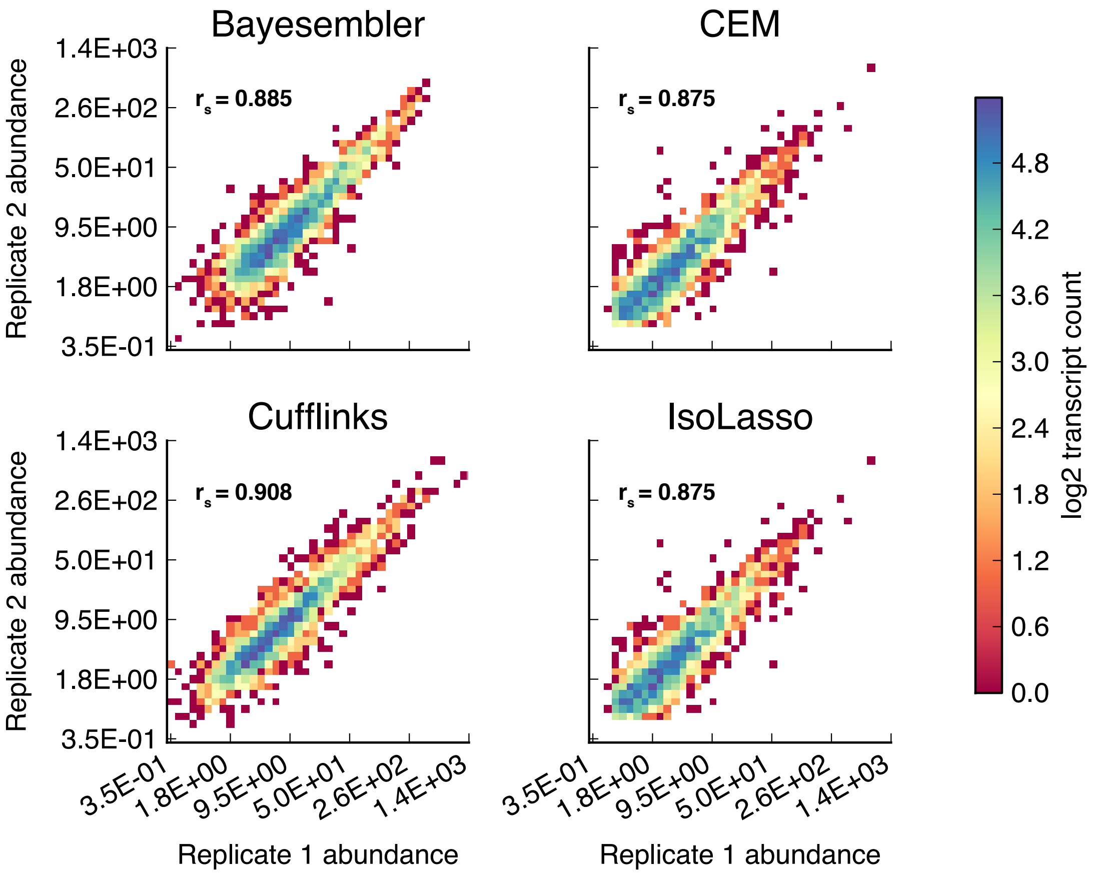

Figure S7: Inter-replicate correspondence of abundance estimates on H1. The figures show the predicted abundances of transcripts assembled in both H1 replicates [3] by all assemblers. r_s denotes the Spearman's rank correlation coefficient.

Supplementary methods

1 A generative model of the RNA-sequencing process

We here present a model for the process of generating a set of paired-end sequencing reads from a set of transcript candidates. Let $F = \{f_1, f_2 \dots f_n\}$ denote a set of n sequenced fragments (i.e. paired-end reads) and let $I = \{1, 2, \dots, i, \dots, n\}$ define an indexset on F. Let $S = \{s_1, s_2 \dots s_m\}$ denote a set of m transcript candidate sequences and let $J = \{1, 2, \dots, j, \dots, m\}$ define an indexset on S. Finally, let $\mathbf{t}$ be a vector of *transcript candidate indices* for the fragments in F such that $t_i \in J$ and $i \in I$. The process of generating a set of fragments F from a set of transcript candidates S then proceeds as follows.

First, a vector of relative abundances $\mathbf{e}$ for the transcripts in S is sampled from its prior distribution $\mathbb{P}(\mathbf{e})$. F is then generated by for each fragment first sampling a candidate index $t \in J$ from $\mathbb{P}(t|\mathbf{e})$ followed by sampling of a fragment f from $\mathbb{P}(f|t)$. By further assuming that fragments are generated independently, this provides the following factorisation of the joint distribution over sets of fragments F, candidate index vectors $\mathbf{t}$ and candidate abundances $\mathbf{e}$

$$\mathbb{P}(F, \mathbf{t}, \mathbf{e}) = \mathbb{P}(\mathbf{e}) \prod_{i=1}^{n} \mathbb{P}(f_i|t_i)\mathbb{P}(t_i|\mathbf{e}) \tag{1}$$

First define $\mathbb{P}(t|\mathbf{e})$ to be the categorical distribution with parameter vector $\mathbf{e}$

$$\mathbb{P}(t|\mathbf{e}) = e_t \tag{2}$$

The other two terms in equation (1) will be derived in the following two sections.

1.1 A prior distribution over transcript candidate abundances

Define the elements of an abundance vector $\mathbf{e}$ to be random variables taking values in $\{0, \mathbb{R}^+\}$. To construct a prior distribution on $\mathbf{e}$, first let $\mathbf{z}$ be a binary vector of length m, which models which transcripts have non-zero abundance (i.e. are expressed). Furthermore, let each element z_j in $\mathbf{z}$ be independent and Bernoulli distributed with parameter π and let each element e_j in an abundance vector $\mathbf{e}$ depend on z_j in $\mathbf{z}$ such that

$$e_j = 0 \text{ if } z_j = 0 \tag{3}$$
$$e_j > 0 \text{ if } z_j = 1 \tag{4}$$

It follows from independence of the individual elements that the prior probability of a binary vector $\mathbf{z}$ is given by

$$\mathbb{P}(\mathbf{z}|\pi) = \pi^{b_{\mathbf{z}}}(1-\pi)^{m-b_{\mathbf{z}}} \tag{5}$$

where $b_{\mathbf{z}} = \sum_{j=1}^{m} z_j$. Next, let $\mathbf{e}^+$ denote the vector of relative abundances for the expressed transcripts such that $e_j = e_k^+$ when $z_j = 1$, where $k = \sum_{l=1}^{j} z_l$ and $\sum_{k=1}^{b_{\mathbf{z}}} e_k^+ = 1$. Finally, let the elements of $\mathbf{e}^+$ be distributed according to the symmetric, Dirichlet distribution with density function

$$\mathbb{P}(\mathbf{e}^+|\mathbf{z},\gamma) = \frac{\Gamma(\gamma b_{\mathbf{z}})}{\Gamma(\gamma)^{b_{\mathbf{z}}}} \prod_{k=1}^{b_{\mathbf{z}}} (e_k^+)^{\gamma-1} \tag{6}$$

where Γ is the gamma function. The prior density of an abundance vector $\mathbf{e}$ is then given by the factorisation

$$\mathbb{P}(\mathbf{e}|\pi,\gamma) = \mathbb{P}(\mathbf{e}^+,\mathbf{z}|\pi,\gamma) = \mathbb{P}(\mathbf{e}^+|\mathbf{z},\gamma)\mathbb{P}(\mathbf{z}|\pi) \tag{7}$$

$$= \frac{\Gamma(\gamma b_{\mathbf{z}})}{\Gamma(\gamma)^{b_{\mathbf{z}}}} \left(\prod_{k=1}^{b_{\mathbf{z}}} (e_k^+)^{\gamma-1} \right) K_{\mathbf{z}_0} \pi^{b_{\mathbf{z}}} (1-\pi)^{m-b_{\mathbf{z}}} \tag{8}$$

where $K_{\mathbf{z}_0} = \frac{1}{1-\mathbb{P}(\mathbf{z}_0)} = \frac{1}{1-(1-\pi)^m}$ with $\mathbf{z}_0$ being the all-zeros vector. The latter term is needed as the Dirichlet distribution is not defined on the simplex of size zero and we thus need to define $\mathbb{P}(\mathbf{z}|\pi) = 0$ and $\mathbb{P}(\mathbf{e}|\mathbf{z},\gamma) = 0$ when $b_{\mathbf{z}} = 0$ to arrive at a valid density function.

1.2 A generative model of the sequencing process of a transcript

A modified version of the model described by Pachter is used to model the sequencing of transcripts corresponding to the indices in $\mathbf{t}$ [6]. More specifically, given a transcript index t, the three-prime position p of the fragment is first sampled from

$$\mathbb{P}(p|t,S,\mu,\sigma) = \frac{\sum_{l=1}^{p} \mathbb{P}(l|\mu,\sigma)}{\sum_{q=1}^{|s_t|} \sum_{l=1}^{q} \mathbb{P}(l|\mu,\sigma)} \qquad \text{s.t. } p \leq |s_t| \tag{9}$$

where $|s_t|$ denotes the length of sequence s_t and $\mathbb{P}(l|\mu,\sigma)$ denotes the Gaussian distribution, which is used to model the length of the sequencing fragments.

Given a three-prime position, a fragment length - and hence a five-prime position - is then sampled from the renormalised fragment length distribution

$$\mathbb{P}(l|p,\mu,\sigma) = \frac{\mathbb{P}(l|\mu,\sigma)}{\sum_{k=1}^{p} \mathbb{P}(k|\mu,\sigma)} \qquad \text{s.t. } l \leq p \tag{10}$$

Finally, a sequenced fragment f is generated conditioned on the sub-sequence specified by t, p and l, and the corresponding observed quality scores q. The quality score at position k in sequencing read one of fragment f is defined as

$$q_{(1,k)} = -10 \log_{10}(\epsilon_{(1,k)}) \tag{11}$$

where ϵ_k designates the probability of a sequencing error at position k [7]. The probability of an error at position k given a quality score is then given by

$$\mathbb{P}(\text{error}|q_{1,k}) = \epsilon_{(1,k)} = 10^{-\frac{q_{(1,k)}}{10}} \tag{12}$$

Let the individual positions in a sequencing read be independent and let the distribution over the three alternative bases given an error be uniform. The joint distribution over positions k in the left read and right reads, r_1 and r_2, respectively, of a fragment f is then given by

$$\mathbb{P}(r_1|t, q_1, S, p, l) = \prod_{k=1}^{|r_1|} \begin{cases} 1 - \mathbb{P}(\text{error}|q_{(1,k)}) & \text{if } r_{(1,k)} = s_{t,(p-l+k)} \\ \frac{\mathbb{P}(\text{error}|q_{(1,k)})}{3} & \text{if } r_{(1,k)} \neq s_{t,(p-l+k)} \end{cases} \tag{13}$$

$$\mathbb{P}(r_2|t, q_2, S, p, l) = \prod_{k=1}^{|r_2|} \begin{cases} 1 - \mathbb{P}(\text{error}|q_{(2,k)}) & \text{if } r_{(2,k)} = s_{t,(p-|r_2|+k)} \\ \frac{\mathbb{P}(\text{error}|q_{(2,k)})}{3} & \text{if } r_{(2,k)} \neq s_{t,(p-|r_2|+k)} \end{cases} \tag{14}$$

where $|r|$ designates the length of the read. The joint distribution over all sequenced positions in a fragment is then given by

$$\mathbb{P}(f|t, q, S, p, l) = \mathbb{P}(r_1|t, q_1, S, p, l)\mathbb{P}(r_2|t, q_2, S, p, l) \tag{15}$$

Finally, a pair of sequenced reads can then be obtained by first sampling whether an error has occurred for each position conditioned on the quality score as given by equation (12). If an error is to be introduced, a substitution is sampled uniformly over the alternative base identities.

1.3 The joint probability distribution

It follows from the preceding sections that the full joint probability distribution is given by the factorisation

$$\begin{aligned} \mathbb{P}(F, \mathbf{t}, \mathbf{l}, \mathbf{p}, \mathbf{e}|Q, S, \mu, \sigma, \pi, \gamma) &= \mathbb{P}(F|Q, S, \mathbf{l}, \mathbf{p}, \mathbf{t})\mathbb{P}(\mathbf{l}|\mathbf{p}, \mu, \sigma)\mathbb{P}(\mathbf{p}|\mathbf{t}, S, \mu, \sigma)\mathbb{P}(\mathbf{t}|\mathbf{e})\mathbb{P}(\mathbf{e}|\pi, \gamma) \\ &= \mathbb{P}(\mathbf{e}|\pi, \gamma) \prod_{i=1}^{n} \mathbb{P}(f_i|t_i, q_i, S, l_i, p_i)\mathbb{P}(l_i|p_i, \mu, \sigma)\mathbb{P}(p_i|t_i, S, \mu, \sigma)\mathbb{P}(t_i|\mathbf{e}) \end{aligned} \tag{16}$$

The corresponding graphical model is shown in figure 5 in the main manuscript.

2 Approximate inference using Gibbs sampling

The objective is to infer the posterior distribution

$$\mathbb{P}(\mathbf{e}, \mathbf{t} | F, Q, S, \mu, \sigma, \pi, \gamma) \tag{17}$$

We can draw samples from this distribution by iteratively drawing samples from the two conditional distributions

$$\mathbb{P}(\mathbf{e} | \mathbf{t}, \pi, \gamma) \tag{18}$$
$$\mathbb{P}(\mathbf{t} | \mathbf{e}, F, Q, S, \mu, \sigma) \tag{19}$$

where the restricted conditioning follows from conditional independences given by factorisation (16). First, the conditional distribution of $\mathbf{e}$ is derived followed by a derivation of the conditional distribution for $\mathbf{t}$.

2.1 Sampling abundance levels

From the definition of $\mathbb{P}(\mathbf{e}|\gamma, \pi)$ in equation (8) it follows that the posterior distribution over $\mathbf{e}$ can be factorised as

$$\mathbb{P}(\mathbf{e}|\mathbf{t}, \pi, \gamma) = \mathbb{P}(\mathbf{e}^{+}, \mathbf{z}|\mathbf{t}, \pi, \gamma) = \mathbb{P}(\mathbf{e}^{+}|\mathbf{z}, \mathbf{t}, \gamma)\mathbb{P}(\mathbf{z}|\mathbf{t}, \pi, \gamma) \tag{20}$$

To derive $\mathbb{P}(\mathbf{z}|\mathbf{t}, \pi, \gamma)$, first note from Bayes' rule

$$\mathbb{P}(\mathbf{z}|\mathbf{t}, \pi, \gamma) = \frac{\mathbb{P}(\mathbf{t}|\mathbf{z}, \gamma)\mathbb{P}(\mathbf{z}|\pi)}{\mathbb{P}(\mathbf{t}|\pi, \gamma)}$$
$$\mathbb{P}(\mathbf{z}|\mathbf{t}, \pi, \gamma) = \frac{\mathbb{P}(\mathbf{t}|\mathbf{z}, \gamma)\mathbb{P}(\mathbf{z}|\pi)}{\sum_{\mathbf{z}' \in Z} \mathbb{P}(\mathbf{t}|\mathbf{z}', \gamma)\mathbb{P}(\mathbf{z}'|\pi)} \tag{21}$$

where Z denotes the set of m long binary vectors excluding the all zeros-vector $\mathbf{z}_0$. Next, let $\mathbf{c}$ be a count-vector of length m such that $c_j = \sum_{i=1}^{n} 1(t_i = j)$. Using, that $\mathbb{P}(\mathbf{z}|\mathbf{t}, \pi, \gamma)$ only depends on $\mathbf{t}$ through $\mathbf{c}$, we can reexpress equation (21) as

$$\mathbb{P}(\mathbf{z}|\mathbf{c}, \pi, \gamma) = \frac{\mathbb{P}(\mathbf{c}|\mathbf{z}, \gamma)\mathbb{P}(\mathbf{z}|\pi)}{\sum_{\mathbf{z}' \in Z} \mathbb{P}(\mathbf{c}|\mathbf{z}', \gamma)\mathbb{P}(\mathbf{z}'|\pi)} \tag{22}$$

To obtain $\mathbb{P}(\mathbf{c}|\mathbf{z}, \gamma)$, first note that it follows from definition (3) and (4) that

$$\mathbb{P}(c_j > 0 | z_j = 0, \gamma) = 0 \text{ for all } z_j \in \mathbf{z} \tag{23}$$

Provided with this notion, let $J^0 \subseteq J$ be the subset of J that contains the indices of the transcripts that have zero counts in $\mathbf{c}$ (i.e. $J^0 = \{j \in J | c_j = 0\}$). Correspondingly, let $J^{+} \subseteq J$ contain the indices of the transcripts that have positive counts in $\mathbf{c}$ (i.e.

$J^+ = \{j \in J | c_j > 0\}$). Using this, define U to be the set of *feasible* binary vectors given by condition (23)

$$U = \{\mathbf{z} \in Z | z_j = 1 \ \ \forall j \in J^+\} \tag{24}$$

Using this condition, it follows that $\mathbb{P}(\mathbf{c}|\mathbf{z}, \gamma)$ can be expressed as

$$\mathbb{P}(\mathbf{c}|\mathbf{z}, \gamma) = \begin{cases} \int_{\mathbf{e}^+} \mathbb{P}(\mathbf{c}|\mathbf{e}^+, \mathbf{z})\mathbb{P}(\mathbf{e}^+|\mathbf{z}, \gamma) \ \ \mathrm{d}\mathbf{e}^+ & \text{if } \ \mathbf{z} \in U \\ 0 & \text{if } \ \mathbf{z} \notin U \end{cases} \tag{25}$$

First, it follows from the definition of $\mathbb{P}(\mathbf{t}|\mathbf{e}^+, \mathbf{z})$ in equation (2) and from conditional independence of the elements in $\mathbf{t}$ given $\mathbf{e}$ that $\mathbb{P}(\mathbf{c}|\mathbf{e}^+, \mathbf{z})$ is equivalent to the multinomial distribution. As $\mathbb{P}(\mathbf{e}^+|\mathbf{z}, \gamma)$ represents the symmetric Dirichlet distribution, it follows that $\mathbb{P}(\mathbf{c}|\mathbf{z}, \gamma)$ is equivalent to the Dirichlet-multinomial distribution. Hence, we have that

$$\mathbb{P}(\mathbf{c}|\mathbf{z}, \gamma) = \begin{cases} \frac{\Gamma(n+1)\Gamma(b_{\mathbf{z}}\gamma)}{\Gamma(n+b_{\mathbf{z}}\gamma)} \prod_{j \in |J^+|} \frac{\Gamma(c_j+\gamma)}{\Gamma(c_j+1)\Gamma(\gamma)} & \text{if } \ \mathbf{z} \in U \\ 0 & \text{if } \ \mathbf{z} \notin U \end{cases} \tag{26}$$

where again $b_{\mathbf{z}} = \sum_{j=1}^{m} z_j$.

To derive a method of sampling from the posterior distribution given by equation (22), first define $\sim$ to be an equivalence relation on binary vectors such that $\mathbf{z}_p \sim \mathbf{z}_q$ when $b_{\mathbf{z}_p} = b_{\mathbf{z}_q}$. Using this, define $Y = U/\sim$ (i.e. U collapsed to its quotient by $\sim$) to obtain the set of *feasible* equivalence classes. The posterior distribution over equivalence classes is then given by

$$\mathbb{P}([\mathbf{z}]|\mathbf{c}, \pi, \gamma) = \begin{cases} \frac{\mathbb{P}(\mathbf{c}|[\mathbf{z}],\gamma)\mathbb{P}([\mathbf{z}]|\pi)}{\sum_{y \in Y} \mathbb{P}(\mathbf{c}|y,\gamma)\mathbb{P}(y|\pi)} & \text{if } \ [\mathbf{z}] \in Y \\ 0 & \text{if } \ [\mathbf{z}] \notin Y \end{cases} \tag{27}$$

From distribution (5) and (26) it follows that $\mathbb{P}(\mathbf{z}|\pi)$ and $\mathbb{P}(\mathbf{c}|\mathbf{z}, \gamma)$ are equal for all $\mathbf{z} \in [\mathbf{z}]$ when $[\mathbf{z}] \in Y$. Using this, the sum over $\mathbf{z} \in [\mathbf{z}]$ can thus be replaced by the cardinality of $[\mathbf{z}] \in Y$. Hence, the posterior distribution over equivalence classes becomes

$$\mathbb{P}([\mathbf{z}]|\mathbf{c}, \pi, \gamma) = \begin{cases} \dfrac{\binom{|J^0|}{b_{[\mathbf{z}]}-|J^+|}\mathbb{P}(\mathbf{c}|\mathbf{z},\gamma)\mathbb{P}(\mathbf{z}|\pi)}{\sum_{y \in Y} \binom{|J^0|}{b_y-|J^+|}\mathbb{P}(\mathbf{c}|\mathbf{z}_y,\gamma)\mathbb{P}(\mathbf{z}_y|\pi)} & \text{if } \ [\mathbf{z}] \in Y \\ 0 & \text{if } \ [\mathbf{z}] \notin Y \end{cases} \tag{28}$$

where $\mathbf{z} \in [\mathbf{z}]$, $\mathbf{z}_y \in y$ and $b_{[\mathbf{z}]}$ and b_y are the simplex sizes defined by $\mathbf{z}$ and $\mathbf{z}_y$, respectively.

From the definition of the equivalence class it follows that a sum over equivalence classes corresponds to a sum over simplex-sizes. By substituting in equations (5) and (26), we can re-express the posterior distribution as

$$\mathbb{P}([\mathbf{z}]|\mathbf{c},\pi,\gamma) = \begin{cases} \frac{\binom{|J^0|}{b_{[\mathbf{z}]}-|J^+|}\frac{\Gamma(b_{[\mathbf{z}]}\gamma)}{\Gamma(n+b_{[\mathbf{z}]}\gamma)}\pi^{b_{[\mathbf{z}]}}(1-\pi)^{m-b_{[\mathbf{z}]}}}{\sum_{b=|J^+|}^{m}\binom{|J^0|}{b-|J^+|}\frac{\Gamma(b\gamma)}{\Gamma(n+b\gamma)}\pi^b(1-\pi)^{m-b}} & \text{if } [\mathbf{z}] \in Y \\ 0 & \text{if } [\mathbf{z}] \notin Y \end{cases} \tag{29}$$

where the correction term $K_{\mathbf{z}_0}$ in equation (5) and multiple terms in equation (26) vanishes as they are independent of $b_{[\mathbf{z}]}$ when $[\mathbf{z}] \in Y$.

Using that all $\mathbf{z} \in [\mathbf{z}]$ has equal probability, we have from distribution (29) that the posterior distribution over binary vectors $\mathbf{z} \in U$ is given by

$$\mathbb{P}(\mathbf{z}|\mathbf{c},\pi,\gamma) = \frac{\frac{\Gamma(b_{\mathbf{z}}\gamma)}{\Gamma(n+b_{\mathbf{z}}\gamma)}\pi^{b_{\mathbf{z}}}(1-\pi)^{m-b_{\mathbf{z}}}}{\sum_{b=|J^+|}^{m}\binom{|J^0|}{b-|J^+|}\frac{\Gamma(b\gamma)}{\Gamma(n+b\gamma)}\pi^b(1-\pi)^{m-b}} \tag{30}$$

By conjugacy of the Dirichlet distribution to the multinomial distribution, we have that the posterior probability of the non-zero expression values $\mathbf{e}^+$ are given by the Dirichlet distribution. Equivalent to the definition of $\mathbf{e}^+$, let $\mathbf{c}^+$ denote the vector of occurences c_j for which $z_j = 1$. The posterior density is then given by

$$\mathbb{P}(\mathbf{e}^+|\mathbf{z},\mathbf{c},\gamma) = \frac{\Gamma(n + b_{\mathbf{z}}\gamma)}{\prod_{k=1}^{b_{\mathbf{z}}}\Gamma(c_k^+ + \gamma)}\prod_{k=1}^{b_{\mathbf{z}}}(e_k^+)^{c_k^+ + \gamma - 1} \tag{31}$$

From equation (29) and (31) it follows that samples from the posterior distribution over expression vectors $\mathbf{e}$ can be obtained using the following hierarchical sampling scheme

1. Sample an equivalence class $[\mathbf{z}]$ given $\mathbf{c}$, π and γ from distribution (29)
2. Sample a binary vector $\mathbf{z}$ uniformly from the equivalence class $[\mathbf{z}]$
3. Sample an expression vector $\mathbf{e}^+$ given $\mathbf{z}$ and $\mathbf{c}$ from distribution (31)

Samples from the Dirichlet distribution in (31) was obtained by transforming variables drawn from the gamma distribution as described by Devroye [8].

2.2 Sampling fragment assignments

From conditional independence of the individual elements in $\mathbf{t}$ given $\mathbf{e}$, we have that

$$\mathbb{P}(\mathbf{t}|\mathbf{e},F,Q,S,\mu,\sigma) = \prod_{i=1}^{n}\mathbb{P}(t_i|\mathbf{e},F,Q,S,\mu,\sigma) \tag{32}$$

Using Bayes' theorem, the posterior probability of an individual fragment-transcript map t is given by

$$\mathbb{P}(t|\mathbf{e}, F, Q, S, \mu, \sigma) = \frac{\mathbb{P}(t|\mathbf{e})\mathbb{P}(F|t, Q, S, \mu, \sigma)}{\sum_{j=1}^{m} \mathbb{P}(j|\mathbf{e})\mathbb{P}(F|j, Q, S, \mu, \sigma)} \tag{33}$$

where $\mathbb{P}(t|\mathbf{e})$ is given by distribution (2) and

$$\mathbb{P}(F|t, Q, S, \mu, \sigma) = \sum_{p=1}^{|s_t|} \mathbb{P}(p|t, S, \mu, \sigma) \sum_{l=1}^{p} \mathbb{P}(l|p, \mu, \sigma)\mathbb{P}(F|t, Q, S, p, l) \tag{34}$$

where $|s_t|$ designates the length of transcript s_t. The individual probabilities in equation (34) are defined in section 1.2.

2.3 Abundance normalisation

The abundance estimates generated by the Gibbs sampler corresponds to the fraction of all paired-end reads a transcript explains. Thus, in order to provide a measure of transcript abundance these values need to be normalised to transcript length. Therefore, define

$$|\tilde{s_j}| = \sum_{q=1}^{|s_j|} \sum_{l=1}^{q} \mathbb{P}(l|\mu, \sigma) \tag{35}$$

to be the *effective length* of transcript s_j, where $|s_j|$ denotes the sequence length of transcript s_j and $\mathbb{P}(l|\mu, \sigma)$ denotes the Gaussian distribution used to model fragment lengths. In addition to transcript length, the abundances is normalised to the total library size $|F|$ in order to make the estimates comparable across different sequencing depths. The relative abundance a_j of transcript s_j is then defined to be

$$a_j = \frac{e_j 10^9}{|\tilde{s_j}||F|} \tag{36}$$

This estimate is used to produce the *FPKM* attribute in the *Bayesembler* output.

3 Hyperparameter estimation

3.1 Fragment length distribution estimation

The standard Gaussian distribution with mean μ and standard deviation σ is used to model the fragment length distribution. The fragment lengths of all paired-end reads mapping uniquely to transcripts at least 2500 nt long from single-transcript graphs were used to estimate the parameters. To minimise the influence of outliers, we used the *median* and *median absolute deviance* (MAD) as estimators of μ and σ, respectively, with σ estimated using $\sigma = MAD * 1.4826$.

3.2 Sparsity estimation

To estimate π, the minimum number of candidates required to explain all paired-end reads is first estimated using a greedy minimum set cover method. Formally, let A be an $n \times m$ binary matrix, where the rows and columns represent paired-end reads and candidate transcripts, respectively, and the values indicate whether a transcript contains a paired-end read or not. Then, let $\mathbf{x}$ denote a binary vector of length m such that $x_j = 1$ for each candidate transcript s_j in the minimum cover. The problem is then given by

$$\arg\min_{\mathbf{x}} \ \mathbf{c}^T\mathbf{x} \quad \text{s.t.} \ A\mathbf{x} \geq \mathbf{a} \tag{37}$$

where $\mathbf{a}$ and $\mathbf{c}$ are n and m long vectors of ones, respectively. Finding the optimal solution for this problem requires evaluating all column combinations, which becomes infeasible for large values of m. For this reason, we used a greedy algorithm, where the transcript containing the largest amount of uncovered reads is chosen at each stage as earlier described by Johnson [9]. Finally, π is estimated using

$$\pi = \frac{\sum_{j=1}^{m} x_j}{m} \tag{38}$$

Supplementary references

[1] Meyer LR, Zweig AS, Hinrichs AS, Karolchik D, Kuhn RM, Wong M, Sloan CA, Rosenbloom KR, Roe G, Rhead B, Raney BJ, Pohl A, Malladi VS, Li CH, Lee BT, Learned K, Kirkup V, Hsu F, Heitner S, Harte RA, Haeussler M, Guruvadoo L, Goldman M, Giardine BM, Fujita PA, Dreszer TR, Diekhans M, Cline MS, Clawson H, Barber GP, et al.: **The UCSC genome browser database: extensions and updates 2013.** *Nucleic Acids Res* 2013, **41**(Database issue): D64–D69

[2] Griebel T, Zacher B, Ribeca P, Raineri E, Lacroix V, Guigó R, Sammeth M: **Modelling and simulating generic RNA-Seq experiments with the flux simulator.** *Nucleic Acids Res* 2012, **40**(20): 10073–10083

[3] Djebali S, Davis CA, Merkel A, Dobin A, Lassmann T, Mortazavi A, Tanzer A, Lagarde J, Lin W, Schlesinger F, Xue C, Marinov GK, Khatun J, Williams BA, Zaleski C, Rozowsky J, Röder M, Kokocinski F, Abdelhamid RF, Alioto T, Antoshechkin I, Baer MT, Bar NS, Batut P, Bell K, Bell I, Chakrabortty S, Chen X, Chrast J, Curado J, et al.: **Landscape of transcription in human cells.** *Nature* 2012, **489**(7414): 101–108

[4] Grabherr MG, Haas BJ, Yassour M, Levin JZ, Thompson DA, Amit I, Adiconis X, Fan L, Raychowdhury R, Zeng Q, Chen Z, Mauceli E, Hacohen N, Gnirke A, Rhind N, di Palma F, Birren BW, Nusbaum C, Lindblad-Toh K, Friedman N, Regev A: **Full-length transcriptome assembly from RNA-Seq data without a reference genome.** *Nat Biotechnol* 2011, **29**(7): 644–652

[5] Au KF, Sebastiano V, Afshar PT, Durruthy JD, Lee L, Williams BA, van Bakel H, Schadt EE, Reijo-Pera RA, Underwood JG, Wong WH: **Characterization of the human ESC transcriptome by hybrid sequencing.** *Proc Nat Acad Sci USA* 2013, **110**(50): E4821–E4830

[6] Pachter L: **Models for transcript quantification from RNA-Seq**. (2011) arXiv 1104.3889v2

[7] Ewing B, Hillier L, Wendl M, Green P: **Base-calling of automated sequencer traces using Phred. II. Error probabilities.** *Genome Res* 1998, **8**(3): 186–194

[8] Devroye L: *Non-uniform random variate generation.* New York: Springer; 1986

[9] Johnson D: **Approximation algorithms for combinatorial problems.** *Journal of Computer and System Sciences* 1974, **9**(3): 256–278
